# Reduced Systemic Levels of IL-10 Are Associated with the Severity of Obstructive Sleep Apnea and Insulin Resistance in Morbidly Obese Humans

**DOI:** 10.1155/2015/493409

**Published:** 2015-04-06

**Authors:** Sonia Leon-Cabrera, Yoaly Arana-Lechuga, Enrique Esqueda-León, Guadalupe Terán-Pérez, Antonio Gonzalez-Chavez, Galileo Escobedo, Javier Velázquez Moctezuma

**Affiliations:** ^1^Unidad de Biomedicina, Facultad de Estudios Superiores-Iztacala, Universidad Nacional Autónoma de México, Avenida de los Barrios 1, Los Reyes Iztacala, 54090 Tlalnepantla, MEX, Mexico; ^2^Departamento de Biología de la Reproducción y Clínica de Trastornos de Sueño, Universidad Autónoma Metropolitana-Iztapalapa, 09340 México, DF, Mexico; ^3^Servicio de Medicina Interna, Hospital General de México, 06726 México, DF, Mexico; ^4^Unidad de Medicina Experimental, Facultad de Medicina, Universidad Nacional Autónoma de México, Hospital General de México, 06726 México, DF, Mexico

## Abstract

Obstructive sleep apnea (OSA) has been related to elevation of inflammatory cytokines and development of insulin resistance in morbidly obese (MO) subjects. However, it is still unclear whether the systemic concentration of anti-inflammatory mediators is also affected in MO subjects directly related to the severity of OSA and level of insulin resistance. Normal weight and MO subjects were subjected to overnight polysomnography in order to establish the severity of OSA, according to the apnea-hypopnea index (AHI). Blood samples were obtained for estimation of total cholesterol and triglycerides, insulin, glucose, insulin resistance, tumor necrosis factor alpha (TNF-*α*), interleukin 12 (IL12), and interleukin 10 (IL-10). Serum levels of IL-10 were significantly lower in MO subjects with OSA than in MO and control individuals without OSA. Besides being inversely associated with serum TNF-*α* and IL-12, decreased IL-10 levels were significantly related to increased AHI, hyperinsulinemia, and insulin resistance. Serum IL-10 is significantly reduced in morbidly obese subjects with severe OSA while also showing a clear relationship with a state of hyperinsulinemia and insulin resistance probably regardless of obesity in the present sample. It may be of potential clinical interest to identify the stimulatory mechanisms of IL-10 in obese individuals with OSA.

## 1. Introduction

Obstructive sleep apnea (OSA) is a condition that threatens health, which is characterized by complete or partial upper airway collapse occurring repeatedly during sleep. OSA is a common health problem affecting 10% of men and 3% of women in the general population, with a considerably high prevalence (~40%) among morbidly obese (MO) individuals [1, 2]. Recurrent episodes of upper airway obstruction, progressive hypoxemia, and sleep fragmentation during OSA have been related to the advent of neural, cardiovascular, and metabolic disturbances mainly in extremely obese subjects [3–5]. Recently, some studies have demonstrated that OSA significantly increases the risk of developing insulin resistance and type 2 diabetes in individuals with morbid obesity [6, 7]. Moreover, it has been reported that the presence of OSA is associated with a significant increase of cardiovascular and cerebrovascular disease in MO individuals [8–11]. Thus, OSA is an important risk factor that contributes to the incidence of cardiovascular and metabolic disease mainly in the human population exhibiting severe levels of obesity [12, 13].

In addition, systemic inflammation has been associated to the presence of OSA. Evidence from animal models and clinical trials have shown that OSA is accompanied by increased levels of proinflammatory mediators including interleukin- (IL-) 1 beta, IL-6, IL-8, tumor necrosis factor alpha (TNF-*α*), and C-reactive protein (CRP) [14–16]. Furthermore, surgical treatment of OSA or the use of continuous positive airway pressure (CPAP) has shown to decrease circulating levels of IL-6, TNF-*α*, and CRP in patients with severe OSA, also reducing metabolic and cardiovascular risk [17–19]. Thus, inflammation seems to be involved in promoting a state of insulin resistance and metabolic dysfunction in patients with OSA [20, 21].

However, although a clear relationship among OSA, morbid obesity, insulin resistance, and systemic inflammation has been repeatedly reported [22–25], it is still unclear whether the anti-inflammatory response also plays a role in the relationship between OSA and metabolic dysfunction. Thus, the main goal of this study was to examine serum levels of IL-10 (a cytokine with potent anti-inflammatory actions) in morbidly obese subjects and its possible association with the degree of OSA and insulin resistance.

## 2. Materials and Methods

### 2.1. Subjects

Adult volunteers (age range 22–55; mean: 37.75 ± 11.5 years) were recruited both at the Sleep Disorders Clinic of the Metropolitan Autonomous University and at the Internal Medicine Department of the General Hospital of Mexico. All of the participants signed a written informed consent, which was previously approved by the institutional review board of the General Hospital of Mexico, which guaranteed that the study was conducted in accordance with the principles described in the Helsinki Declaration. Subjects included in the study were healthy controls with a body mass index (BMI) between 18.5 and 25.9 kg/m^2^, as well as morbidly obese subjects (BMI > 40 kg/m^2^). None of the subjects included in the study had previously received a diagnosis or treatment for OSA. Subjects were excluded from the study when they had a previous or recent diagnosis of diabetes mellitus, chronic hepatic or renal disease, and blood pressure higher than 140/90 mm Hg, inflammatory or autoimmune disorders, acute or chronic infectious diseases, cancer, and endocrine disorders. Additionally, we excluded pregnant or lactating women, subjects taking anti-inflammatory, antiaggregant, and antihypertensive drugs, and subjects that had not fasted overnight for 8 hours. All of the morbidly obese patients took 20 mg/day atorvastatin at the time of being included into the study. All of the individuals enrolled in the study received a full medical evaluation, which included taking their clinical history and a physical examination by qualified physicians.

### 2.2. Questionnaire

All of the subjects included in the study completed a questionnaire developed in the Sleep Disorders Clinic of the Metropolitan Autonomous University, about their normal sleep routine, symptoms of sleep-disordered breathing and family history. These questionnaires were also used to assess eligibility for the study according to the previous inclusion criteria.

### 2.3. Polysomnographic Assessment

Sleep recordings were obtained at the Sleep Disorders Clinic, using a 32 channel Cadwell digital polygraph. Sleep recording included fully conventional EEG recording (32 electrodes, with 16 channels), electrooculography (EOG), chin and tibial electromyography (EMG), and electrocardiographic (EKG) recordings, as well as respiratory variables such as thoracic and abdominal respiratory movements, nasal flow, and oximetry. Sleep stages were scored according to 2007 American Association of Sleep Medicine guidelines for the scoring of sleep and associated events [26]. Respiratory events were derived primarily from the nasal cannula pressure transducer. The oxygen desaturation index (ODI) was defined as the total number of episodes of oxyhemoglobin desaturation ≥3% from the immediate baseline, ≥10 s but <3 min, divided by the total sleep time. Apneas were defined as decrements in airflow ≥90% from baseline for ≥10 s. Hypopneas were defined as 30% or greater decrease in flow lasting at least 10 s and associated with a 4% or greater oxyhemoglobin desaturation. The number of apneas and hypopneas per hour of sleep was calculated to obtain the apnea-hypopnea index (AHI). The severity of OSA was assessed according to the AHI values, a value of more than 15 was considered as moderate to severe OSA. In our study population, hypoventilation syndrome was excluded by verifying that subjects had saturation oxygen (SaO_2_) greater than 90% and partial pressure of carbon dioxide (PaCO_2_) less than 45 mmHG at the vigil. SaO_2_ was measured by digital oximetry (Healthdyne Technologies) while PaCO_2_ was assessed by capnography (CO_2_ Meter, USA).

### 2.4. Anthropometric Measurements

Height and weight measurements were recorded from each subject to calculate the body mass index (BMI). According to both the BMI and the AHI, all of the participants were divided into three groups: control nonobese subjects (BMI 18.5–24.9 kg/m^2^, **N** = 10) without OSA, morbidly obese subjects (BMI ≥ 40 kg/m^2^, **N** = 13), and morbidly obese subjects (BMI ≥ 40 kg/m^2^, **N** = 29) with a diagnosis of OSA. Waist circumference was also obtained from each study subject, always measuring at the midpoint between the lower rib margin and the iliac crest using a conventional measuring tape in centimeters (cm).

### 2.5. Biochemical Measurements

Blood samples were individually obtained after an 8-h overnight fast and collected into pyrogen-free tubes (Vacutainer, BD Diagnostics, NJ, USA) at room temperature. Collection tubes were then centrifuged at 1000 g/4°C for 30 min, and serum samples were obtained and stored at −80°C in numerous aliquots until the time of use. Total cholesterol and triglycerides were individually measured in triplicate by enzymatic assay according to the manufacturer's instructions (Randox, Mexico). Serum insulin levels were individually determined in triplicate by means of the Enzyme-Linked Immunosorbent Assay (ELISA), following the manufacturer's instructions (Abnova Corporation, Taiwan). Serum glucose levels were individually determined in triplicate by the glucose oxidase assay, following the manufacturer's instructions (Randox, Mexico). All of the biochemical measurements were performed at the same time in order to avoid procedural variations. The estimate of insulin resistance was determined in each study subject by means of the Homeostatic Model Assessment-Insulin Resistance (HOMA-IR) calculation, as follows: fasting insulin concentration (mU/L) × fasting glucose concentration (mmol/L) divided by 22.5 [27].

### 2.6. Cytokine Measurements

Serum levels of TNF-*α*, IL-12, and IL-10 were individually determined in triplicate by ELISA, following the manufacturer's instructions (Peprotech, Mexico). The experimental measurement of IL-10 was performed at the same time in order to avoid procedural variations.

### 2.7. Statistical Analyses

Data regarding age, BMI, waist circumference, percentage of body fat, fasting serum glucose, fasting serum insulin, HOMA-IR, total cholesterol and triglycerides, and polysomnographic parameters were analyzed as mean ± standard deviation. One-way ANOVA followed by a post hoc Tukey test were performed to determine significant differences in the aforementioned parameters. A Chi-squared test was performed to estimate differences in frequency variables (male/female proportion). Data from IL-10 were analyzed as the median in interquartile ranges using box plot analysis. The Kruskal-Wallis test, followed by Dunn's multiple comparison test, was performed to determine significant differences concerning the levels of IL-10, IL-12, and TNF-*α*. The Spearman's correlation coefficient was performed to assess the statistical association of IL-10 and AHI with anthropometric, biochemical, and inflammatory parameters. All of the statistical analyses were performed using the GraphPad Prism 5 software. Differences were considered significant when *P* < 0.05.

## 3. Results

Table 1 shows the results obtained. A total of 52 subjects were included in the study, 10 were normal weight controls, 13 were morbidly obese individuals, and 29 were morbidly obese subjects with polysomnographically diagnosed OSA. No significant differences were observed in the mean age among all of the groups studied. In terms of anthropometric and metabolic data, the BMI, abdominal circumference, fasting blood glucose, and total triglycerides showed a significant increase in both MO and MO+OSA groups as compared to controls. However, no significant differences were observed in the aforementioned metabolic parameters between MO and MO+OSA individuals. Both serum insulin and HOMA-IR values were significantly higher in MO+OSA subjects when compared to controls and MO subjects. Total cholesterol levels did not show any difference among any of the groups studied but it should be probably related to the statin therapy. Concerning inflammatory parameters, serum levels of IL-12 were significantly higher in both MO groups when compared to controls but no differences were observed when both MO groups were contrasted. TNF-*α* showed a significant increase in the MO+OSA individuals when compared to controls. MO subjects showed no significant differences compared to controls and when both MO groups were contrasted no significant differences in TNF-*α* were observed.

Polysomnographic results are summarized in Table 2. The mean AHI value was 7-fold higher in MO+OSA individuals than in MO and control subjects (for the MO+OSA group 51.4 ± 25.7, for the MO group 7.25 ± 3.4, and for the control group 7.5 ± 3.3). Thus, all of the individuals categorized into the MO+OSA group exhibited a moderate to severe degree of obstructive sleep apnea. Sleep latency was significantly longer in both MO subjects, whereas the percentage of rapid eye movement (REM) sleep showed a significant decrease in MO+OSA patients compared to control subjects. No significant differences were observed in the values of total sleep time, sleep efficiency percentage, light sleep percentage, slow wave sleep percentage, and mean SaO_2_ among all of the groups studied (Table 2).

**Figure 1:**
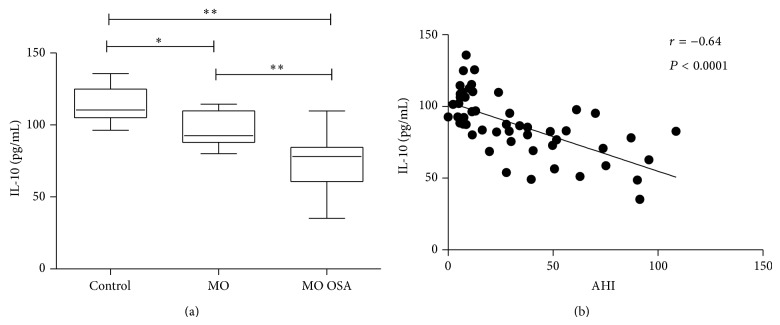
(a) Box plot of circulating IL-10 levels for control, MO, and MO+OSA subjects. Data are expressed as the median and interquartile range in a box plot analysis. (b) Correlation between AHI and IL-10 circulating levels. AHI was negatively correlated with IL-10 levels. Coefficient *r* and *P* values were calculated by the Spearman's correlation model. The correlation level was considered significant when *P* < 0.05. ^∗^ *P* < 0.05 and ^∗∗^ *P* < 0.001.

Concerning IL-10, systemic levels exhibited a significant decrease in morbidly obese subjects when compared to controls. However, the serum values of this anti-inflammatory cytokine were even more significantly reduced when morbid obesity was accompanied by obstructive sleep apnea (Table 1). The mean value of IL-10 in normal weight controls and MO individuals was 113.20 ± 12.10 and 97.20 ± 10.90 pg/mL, respectively, whereas in MO+OSA subjects it was 74.40 ± 17.0 pg/mL (Table 1, Figure 1(a)).  Figure 1(b) shows the correlation between serum levels of IL-10 and AHI. A significant inverse correlation was observed. IL-10 decrease was inversely related to the OSA severity. Higher levels of AHI correspond with the lower levels of IL-10 (*r* = −0.64, *P* < 0.0001).

**Figure 2:**
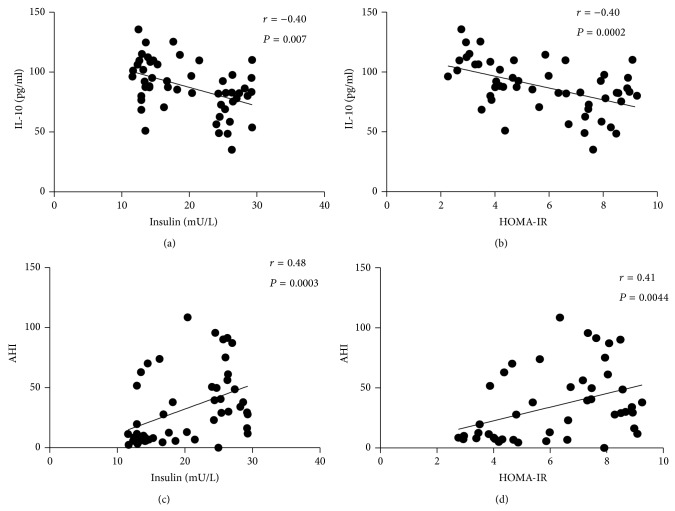
Circulating levels of IL-10 showed a negative significant association with (a) insulin levels and (b) HOMA-IR index. In contrast, AHI values were positively associated with (c) Insulin levels and (d) HOMA-IR index. Coefficient *r* and *P* values were calculated using Spearman's correlation model. The correlation level was considered significant when *P* < 0.05.

As was mentioned above, MO+OSA individuals exhibited a state of hyperinsulinemia and insulin resistance when compared with MO and control subjects (Table 1). Thus, to further analyze the association of IL-10 with these parameters, a correlation analysis was performed between IL-10 and both insulin and insulin resistance.  Figure 2(a) shows a significant inverse correlation between IL-10 and blood insulin levels (*r* = −0.4; *P* = 0.007). As IL-10 levels decrease, insulin levels increase. A quite similar picture is observed in Figure 2(b). A significant inverse correlation between IL-10 levels and the insulin resistance index is shown (*r* = −0.40; *P* = 0.0002). As IL-10 decrease the HOMA-IR increases. To further analyze the relationship between insulin and the presence of AHI a correlation analysis was performed. As can be seen in Figures 2(c) and 2(d), there is a significant positive correlation between AHI and insulin (*r* = 0.48; *P* = 0.0003), as well as a positive correlation between AHI and HOMA-IR (*r* = 0.41; *P* = 0.004). The severity of apnea correlates with the increase of insulin and the increase of insulin resistance.

In addition, significant correlations were found between the decrease in IL-10 and some sleep parameters, such as sleep latency (*r* = −0.53, *P* = 0.0092), light sleep percentage (*r* = −0.38, *P* = 0.033), and REM sleep percentage (*r* = 0.49, *P* = 0.0083) (Table 3).

## 4. Discussion

Present results show that serum levels of the anti-inflammatory cytokine IL-10 are significantly reduced in morbidly obese subjects with obstructive sleep apnea and show a strong correlation with a systemic state of hyperinsulinemia and insulin resistance.

A growing body of evidence has now suggested that the occurrence of metabolic dysfunction in obese subjects not only may be favored by obesity itself but also may be due to obesity-related comorbidities such as obstructive sleep apnea [28]. In fact, a recent study performed in individuals with less than 6 hours of sleep reported that the presence of OSA is more importantly related to the development of hyperglycemia and hyperinsulinemia than obesity [29]. Similarly, in Caucasians it has been described that the level of insulin resistance is associated with the severity of OSA, regardless of the patient's BMI [30]. An additional study performed in Latin Americans showed a strong association between OSA and an altered glucose and lipid metabolism, irrespective of other confounding factors including age, gender, and obesity [31]. It has been also shown that the HOMA-IR value increased 0.5% with each additional apnea or hypopnea event per sleeping hour, suggesting a direct association between OSA and insulin resistance [32].

Consistent with this information, our study population did not show an altered response to insulin despite having morbid obesity. However, when extreme obesity was accompanied by moderate or severe obstructive sleep apnea, the insulin resistance level started to increase reaching two hundred percent higher than in normal conditions (HOMA-IR ≤ 3.80 as the criterion for noninsulin resistance for Mexicans) [33]. In light of this information, it is clear that obstructive sleep apnea is not simply a comorbidity associated with obesity but a sleep disorder, possibly contributing to the development of insulin resistance and other metabolic dysfunctions, probably regardless of obesity.

Systemic inflammation has been recently shown to be associated with OSA and metabolic disturbances [8, 10]. In fact, some studies have reported abnormally high circulating levels of inflammatory cytokines including IL-6, IL-8, IFN-*γ*, and TNF-*α* in obese individuals exhibiting OSA and insulin resistance/type 2 diabetes [29, 30]. Evidence from obese mouse models has demonstrated that hypoxia (a common feature in OSA) is capable of promoting the production of inflammatory factors [34]. Studies in obese humans and animals suggest that hypoxia resulting from recurring apneas during OSA may contribute to the activation of transcription factors including nuclear factor kappa B (NF-*κ*B) and the hypoxia inducible factor 1*α* (HIF-1*α*) [35]. In hypertrophic adipose tissue (a frequent characteristic of obesity), NF-*κ*B and HIF-1*α* are capable of overregulating the expression of genes that encode proinflammatory cytokines, such as IL-6 and TNF-*α* which, in turn, have direct deleterious effects on the insulin cell signaling pathway [36]. Thus, obesity and OSA-related hypoxia have been proposed as main promoting factors of a systemic inflammatory response in obese patients with OSA and metabolic disease [20, 21]. However, although there is growing evidence pointing to the importance of systemic inflammation in the development of metabolic abnormalities, it has been difficult to determine whether the increase of proinflammatory factors is preferentially related to obesity rather than OSA and* vice versa* [28, 37]. In this sense, our results clearly indicate a systemic inflammatory state in extremely obese subjects, characterized by high circulating levels of TNF-*α* and IL-12. However, such an increase in the aforementioned inflammatory parameters was observed when comparing morbidly obese subjects with normal weight controls and not when comparing morbidly obese individuals* versus* OSA morbidly obese patients. In other words, elevation of the serum levels of TNF-*α* and IL-12 in our study population seems to be preferentially related to the fat mass gain rather than the occurrence of obstructive sleep apnea and insulin resistance.

It is still unclear whether the systemic inflammatory scenario is only due to increased production of proinflammatory cytokines or is also influenced by a decrease of anti-inflammatory cytokines. In this study, serum levels of the anti-inflammatory cytokine IL-10 showed a 15% reduction related to obesity itself (Controls* versus* MO) and a 35% decrease associated with OSA regardless of obesity (MO* versus* MO+OSA). In fact, there was a significant negative correlation between serum IL-10 and the index of apneas/hypopneas in the morbid obesity group; in other words, our results suggest that OSA severity increases while serum levels of IL-10 decrease. Accordingly, a recent study showed that serum levels of TNF-*α* and TNF-*α*/IL-10 ratio are increased in patients with severe OSA, while circulating values of IL-10 are significantly decreased with respect to controls [38]. These data show that increasing in inflammatory mediators during OSA is usually accompanied by a reduction in the circulating levels of anti-inflammatory cytokines, an inverse association that could be useful for assessing and monitoring OSA in patients. However, further clinical prospective studies are still needed to determine whether decreasing in anti-inflammatory factors could be secondary to systemic inflammatory activation in a causative fashion.

A relatively recent study revealed that inverse relationship between increased production of inflammatory markers and decreased levels of anti-inflammatory mediators could appear not only until severe OSA but also since initial stages of such sleep disorder [39]. In our study population, normal weight controls and MO subjects exhibited a mild grade of OSA (AHI 7.5 ± 3.3 and 7.25 ± 3.4, resp.). However, inflammatory/anti-inflammatory cytokine pattern was clearly dissimilar between mild and severe OSA groups. As previously mentioned, increased systemic values of IL-12 were seen in both MO and MO+OSA groups with respect to normal weight controls, regardless of OSA severity and perhaps preferentially associated with obesity degree. In contrast, TNF-*α* exhibited higher levels in patients with severe OSA than in subjects exhibiting mild OSA; nevertheless, no significant differences were observed in mild OSA individuals despite of having clear differences in terms of BMI. Notably, IL-10 showed to be a more specific marker to differentiate between severe and mild OSA patients. IL-10 exhibited reduced circulating levels in severe OSA patients with respect to mild OSA subjects, even between individuals showing morbid obesity. Thus, inflammatory/anti-inflammatory imbalance appears to become evident since initial stages of OSA, interestingly, when hypoxia is not so severe. It is then of great importance to determine what factors could be involved in increasing the risk to develop a more robust inflammatory response in OSA, even in the absence of severe hypoxia. In this sense, a growing body of evidence has now suggested augmented risk to develop OSA in subjects having a single nucleotide polymorphism (SNPs) in the TNF-*α* gene promoter region (TNF-*α*-308G/A), which may be directly associated with increased TNF-*α* production [40, 41]. Therefore, further investigations are still needed to identify SNPs in IL-10 and other inflammatory/anti-inflammatory cytokines with the aim to determine whether such markers could be used for individually evaluating the risk to develop OSA, even at early stages and regardless of obesity. Besides the possible role of genetic susceptibility, it is important to determine other factors potentially involved in reducing IL-10 and thus increase the risk to develop OSA. In this sense, OSA patient-derived gamma/delta T cells show increased intracellular contents of the proinflammatory cytokines TNF-*α* and IL-8 but decreased synthesis of IL-10, suggesting a reduced capacity of T cells to produce IL-10 during OSA [42]. Furthermore, in a previous study conducted in nonobese children, circulating IL-10 levels were lower in OSA children than in control subjects. Interestingly, the levels of IL-10 were shown to be restored to normal values after subjecting these patients to surgical adenotonsillectomy, which suggest that narrowing of the airway during sleep may associate with hypoxia and interruption of IL-10 synthesis [43]. As previously mentioned, hypoxia has been demonstrated to play a pivotal role in NF-*κ*B activation [35]. Besides being related to IL-12 and TNF-*α* production, NF-*κ*B has been shown to downregulate IL-10 synthesis in C57BL/6J mice [44]. Such notable information suggests that NF-*κ*B may be involved in the diminution of IL-10, which highlights the importance to take into account the individual capacity of obese patients to activate NF-*κ*B-dependent signaling pathways and thus increase the risk to develop OSA. For this reason, it is extremely important to perform clinical studies addressed not only to evaluate the levels of inflammatory/anti-inflammatory cytokines in OSA patients but also to examine intracellular signaling pathways directly involved in cytokine production.

Besides, increased insulin was observed in morbidly obese subjects with obstructive sleep apnea but not in morbidly obese individuals without such a sleep-related disorder. We then corroborated that most of the subjects showing low systemic levels of IL-10 and high AHI value also had increased insulin resistance, suggesting a direct association among OSA, anti-inflammatory factors, and metabolic abnormalities irrespective of obesity. IL-10 is a cytokine with anti-inflammatory properties capable of modulating inflammatory responses by suppressing the production of proinflammatory cytokines (IL-1*β*, TNF-*α*, IL-6, and IL-8) [45]. Low plasma levels of IL-10 have been associated with an increased risk of insulin resistance and type 2 diabetes in obese subjects, while the occurrence of the above-mentioned metabolic alterations has been shown to be favored by OSA [28, 29, 46]. However, obesity frequently concurs with both obstructive sleep apnea and obesity, which has made it difficult to determine whether IL-10 could be specifically altered during OSA and thus contribute to metabolic disease regardless of obesity. Moreover, additional studies have also pointed out to other obesity-related metabolic abnormalities that may influence cytokine levels, as is the case of hypertension. Indeed, OSA patients with hypertension show reduced values of IL-10 accompanied by increased levels of TNF-*α*, IL-6, and CRP with respect to patients without high blood pressure [47]. Similarly, stroke patients with severe OSA exhibit increased levels of serum IL-6 when they also show hypertension [48]. These data suggest that hypertension may associate with decreased production of anti-inflammatory factors but increased levels of inflammatory mediators. Thus, it is really important to take into consideration the potential contribution of other obesity-related abnormalities on the inflammatory/anti-inflammatory response in OSA patients.

## 5. Conclusions

In conclusion, the present study demonstrated that serum levels of the anti-inflammatory cytokine IL-10 are significantly reduced in morbidly obese subjects with obstructive sleep apnea and show a strong correlation with a systemic state of hyperinsulinemia and insulin resistance. These results suggest an obesity-independent relationship among OSA, decreased IL-10, and the development of insulin resistance. It is still necessary to augment the size of the study population having a similar male/female proportion in order to understand the relationship among OSA, metabolic dysfunction, and IL-10 in a gender-specific fashion. Also, additional studies including normal weight subjects with an OSA diagnosis are of great importance in order to accurately determine whether IL-10 may be useful as a marker of increased risk for OSA and insulin resistance, even in the absence of obesity.

## Figures and Tables

**Table 1:** Anthropometric, biochemical, and immunological characteristics of the study subjects.

	Control subjects *n* = 10	MO *n* = 13	MO+OSA *n* = 29	Control versus MO	Control versus MO+OSA	MO versus MO+OSA
Male proportion (%)	8 (80)	2 (15)	4 (13)	0.0018	0.0002	>0.9
Age (years)	43.4 ± 11.5	33.5 ± 10.9	37.2 ± 11.4	0.09	0.3	>0.9
Body mass index (kg/m^2^)	23.6 ± 2.1	45.4 ± 8.2	45.2 ± 8.4	<0.0001	<0.0001	>0.9
Abdominal circumference (cm)	84.3 ± 6.7	129.9 ± 21.9	124.2 ± 16.4	<0.0001	<0.0001	>0.9
Fasting blood glucose (mmol/L)	5.0 ± 0.5	6.87 ± 0.27	6.7 ± 0.57	<0.0001	<0.0001	0.8
Fasting blood insulin (mU/L)	13.5 ± 1.8	17.4 ± 5.1	22.9 ± 5.6	0.16	<0.0001	0.0052
HOMA-IR	3 ± 0.4	5.3 ± 1.6	6.9 ± 1.8	0.0042	<0.0001	0.01
Total cholesterol (mg/dL)	192.3 ± 36.1	198.8 ± 29	201.6 ± 46.1	0.9	0.8	0.9
Total triglyceride (mg/dL)	121.8 ± 37.4	215.8 ± 59.1	227.3 ± 62.8	0.0008	<0.0001	0.8
IL-12 (pg/mL)	217.4 ± 43	394 ± 64.7	405.3 ± 57.4	0.0002	<0.0001	0.9
TNF-*α* (pg/mL)	270.2 ± 31.7	306.9 ± 38.9	337.9 ± 67.8	0.12	0.0031	0.8
IL-10 (pg/mL)	113.2 ± 12.10	97.2 ± 10.9	74.40 ± 17.0	0.36	<0.0001	0.0012

HOMA-IR, homeostatic model assessment-insulin resistance; AHI, apnea-hypopnea index; TNF-*α*, tumor necrosis factor alpha; IL, interleukin; NS, nonsignificant differences. Data are presented as mean ± standard deviation. Differences were considered significant when *P* < 0.05.

**Table 2:** Polysomnographic characteristics of the study subjects.

	Control	MO	MO+OSA	Control versus MO	Control versus MO+OSA	MO versus MO+OSA
Total sleep time (hours)	7.3 ± 0.5	6.7 ± 0.93	6.6 ± 0.4	>0.9	>0.9	>0.9
Sleep latency (min)	7.5 ± 4.6	26.3 ± 17.6	42.1 ± 28.8	0.0054	0.0054	>0.9
Sleep efficiency (%)	89.6 ± 6.4	83.5 ± 10.8	81.6 ± 9	>0.9	>0.9	>0.9
Light sleep (%)	61.6 ± 9.5	63 ± 5.4	72.6 ± 10	>0.9	>0.9	>0.9
Slow wave sleep (%)	17.6 ± 8	19.5 ± 2	15.4 ± 10	>0.9	>0.9	>0.9
REM sleep (%)	26.4 ± 18.7	17.5 ± 5	12 ± 4.5	>0.9	0.0095	>0.9
Mean SaO_2_	90.7 ± 1	93.2 ± 1	84.7 ± 10.8	>0.9	>0.9	>0.9
Total arousal index	7.37 ± 2.5	6.25 ± 3.5	28.53 ± 21	>0.9	<0.01	<0.01
SaO_2_ < 90%	2.45 ± 1.08	1.56 ± 1.09	39.40 ± 13.03	<0.001	<0.001	>0.9
AHI (events/hour)	7.5 ± 3.3	7.25 ± 3.4	51.4 ± 25.7	>0.9	<0.001	<0.001

Data are presented as mean ± standard deviation. Differences were considered significant when *P* < 0.05. AHI, apnea-hypopnea index.

**Table 3:** Correlation between circulating levels of IL-10 and polysomnographic characteristics of the study subjects.

	IL-10 *r*	*P* value
Total sleep time (hours)	0.24	NS
Sleep latency (min)	−0.53	0.0092
Sleep efficiency (%)	0.19	NS
Light sleep (%)	−0.38	0.033
Slow wave sleep (%)	0.14	NS
REM sleep (%)	0.49	0.0083
Mean SaO_2_	0.18	NS
Total arousal index	−0.6	0.0065
AHI	−0.64	<0.0001

Coefficient *r* and *P* values were calculated by the Spearman's correlation model. The correlation level was considered significant when *P* < 0.05. AHI, apnea-hypopnea index.
